# Clinical characteristics and favorable long-term outcomes for patients with idiopathic inflammatory myopathies: a retrospective single center study in China

**DOI:** 10.1186/1471-2377-11-143

**Published:** 2011-11-09

**Authors:** Xiao Ming Shu, Xin Lu, Yao Xie, Guo Chun Wang

**Affiliations:** 1 Department of Rheumatology, China-Japan Friendship Hospital, the Ministry of Health, Ying Hua East Road, Chao Yang District, 100029, Beijing, China

## Abstract

**Background:**

Little is known about the clinical features and true survival risk factors in Chinese Han population. We conducted the current study to investigate the clinical features, long-term outcome and true potential indicators associated with mortality of idiopathic inflammatory myopathies (IIM) in China.

**Methods:**

We restrospectvely investigated 188 patients diagnosed with IIM at our hospital from January 1986 to April 2009. The primary outcome was determined with mortality. The secondary outcomes for survival patients were organ damage and disease activity, health status, and disability, which were assessed with Myositis Damage Index, Myositis Disease Activity Assessment Visual Analogue Scales, Health Assessment Questionnaire Disability Index, and the Modified Rankin Scale, respectively. Potential prognostic factors for mortality were analyzed with the multivariate Cox regression model.

**Results:**

Mean age at disease onset was 43.8 ± 15.8 years and male to female ratio was 1:2.1 in this cohort. The 1-, 5-, 10-, 15- and 20-year survival rates were 93.6%, 88.7%, 81%, 73.6% and 65.6%. The independent predicators for mortality were age at disease onset [hazard ratio (HR):1.05, 95% CI 1.02 - 1.08], presence of cancer (HR:3.68, 95%CI 1.39 - 9.74), and elevated IgA level at diagnosis (HR:2.80, 95% CI 1.16-6.74). At the end of the follow-up, 29 patients manifested drug withdrawal within an average 4.1 years (range 0.5-15.2 year), most patients (85.9%) had no disease activity and 130 patients (83.4%) had no disability.

**Conclusions:**

The long-term outcomes of IIM patients in our cohort have improved dramatically. Those patients most likely to survive had a high chance of reaching stable disease status, and obtained long-term or possibly permanent remission to a large extent.

## Background

The idiopathic inflammatory myopathies (IIM) are a group of systemic diseases that affect primarily muscle and skin, but also may affect many other organs, such as the lungs, heart, joint and gastrointestinal tract [1,2]. They can be classified into polymyositis (PM), dermatomyositis (DM), juvenile polymyositis or dermatomyositis (JM), amyophathic dermatomyositis (ADM), overlap myositis (OM), and inclusion body myositis (IBM) [3-7].

Numbers of survival analyses performed among IIM patients in Caucasian populations have shown that 5-year survival ranges from 52%-95% [1,8-14]. Moreover, previous studies have displayed many predictors of poor outcome such as old age, male gender, delay in diagnosis and therapy, associated malignancy, fever, anti-Jo-1 positive, and the presence of complications (dysphagia, dysphonia, pulmonary, and cardiac involvement) [8,9,12-16]. However, there are disagreements among these studies, especially regarding the role of ethnicity, clinical manifestation and treatment.

Although it has been suggested that the mortality of IIM has been greatly reduced, many IIM patients continue to exhibit physical disability and organ damage [1,2,17-21]. However, long-term follow-up studies of organ damage assessed through standard assessment tools have been limited. Furthermore, formerly published survival data mainly represent mortality and morbidity in non-Chinese Han populations. So far, very limited data on clinical characteristics, long-term outcome, and prognostic factors are available for patients with IIM in the Chinese Han population.

These findings prompted us to assess the clinical characteristics and long-term outcome of Chinese patients with IIM through using the standard assessment tools; and to evaluate possible prognostic factors for mortality.

## Methods

### Patients

We retrospectively reviewed the medical records of 188 patients with IIM excluded inclusion body myositis which had full clinical information and in our cohort myositis center (Department of Rheumatology in China-Japan Friendship Hospital) between January 1986 and April 2009. The Rheumatology center in the China-Japan Friendship Hospital is one of the most famous centers with a particular interest in the disease, and most patients with IIM referred in our center. Therefore, our series represent most of the types of IIM except for juvenile myositis. Because of the Chinese medical policy, the adult patient hospitals were not permitted to be responsible for the health provision of children. The diagnosis of IIM was based on the Bohan and Peter criteria for PM/DM and Sontheimer's definitions for ADM [22,23]. The cases were divided into the following subsets: (i) adult PM and DM; (ii) juvenile PM/DM (JM) with age < 18 years at diagnosis; (iii) ADM; (iv) cancer associated with PM/DM; and (v) overlap myositis (myositis in combination with other connective tissue disease). Cases which were not Chinese Han population, or lost to follow-up, or IBM were excluded.

Informed consents were obtained from all patients or parents. This study was approved by the ethical committee of the China-Japan Friendship Hospital.

### Data collection from clinical charts

Data on history, physical and laboratory findings were obtained by retrospective medical record review using a standardized protocol. At the time of visiting to our center, all patients underwent detailed clinical examination including extramuscular (interstitial lung disease, cardiac involvement, dysphagia, pulmonary arterial hypertension and cancer), laboratory tests (including autoantibodies), and electromyography (EMG). During the clinical course of the disease, these tests were usually conducted as required.

Disease activity at disease onset was assessed by the Myositis Disease Activity Assessment Visual Analogue Scales (MYOACT) established by the International Myositis Assessment and Clinical Studies (IMACS) Group according to patients' charts [24].

Definitions for target organ involvement were as follows: (i) Interstitial lung disease (ILD) was diagnosed if chest radiograph and high resolution computed temography (HRCT) scan indicated the presence of parenchymal micronodules and nodules, linear opacities, irregularity of the interfaces between peripheral pleura and aerated lung parenchyma, ground-glass opacities, honeycombing, and traction bronchiectases or bronchiolectases, and isolated restrictive pattern on pulmonary function tests [25]. (ii) cardiac involvement was defined on the basis of new onset of rhythm disturbances, conduction abnormality, cardiomyopathy, myocarditis, or congestive heart failure during IIM disease course after ruling out coronary artery disease and other cause of cardiac diseases such as hypertension, rheumatic heart disease, congenital heart disease, or diabetes. The diagnostic tools for cardiac involvement were detected using electrocardiography (ECG) or echocardiography, and biochemistry test including cardiac isoform troponin-I (cTnI), B type natriuretic peptide (BNP), Creatine kinases (CK) and CK-MB. (iii) Pulmonary arterial hypertension (PAH) was diagnosed if systolic pulmonary arterial pressure was greater than 30 mmHg at rest on doppler echocardiography. (iv) Dysphagia was determined by dysphagia symptoms, gastroesophageal reflux, marked decrease of peristalsis or barium persistence checked by upper gastrointestinal tract visualization.

### Follow-up assessment

After the data collection from patient charts, we interviewed each patient by telephone (50/188, 26.6%) or face to face (138/188, 73.4%) at the outpatient clinic during May 2010, and collected data of main and secondary outcomes. Follow-up period was defined from initial diagnosis to the date of death or to the end of follow-up (May 31, 2010). The main outcome was death observed; the secondary outcomes for survival patients were organ damage and disease activity, health status, and disability, which were assessed with Myositis Damage Index (MDI) [17,19], MYOACT, Health Assessment Questionnaire Disability Index for adult or child (HAQDI range 0-3, where 0 indicates no difficulty with and 3 unable to perform daily activities) [17,18], and the Modified Rankin Scale (MRS) (score 0: no disability; score 3: totally dependent) [20], respectively. In addition, the following clinical assessments were undertaken at the end of follow-up: treatment status of survival patients; and causes of death, which were evaluated from the patient charts when a patient died in our hospital, or obtained from family members when the death occurred outside the hospital.

### Assessment of prognostic factors

The following prognostic factors for mortality were analyzed: age at disease onset; sex; clinical classification of IIM; disease duration from disease onset to diagnosis; myalgia, skin ulcers, typical rashes, presence of Raynaud's phenomenon, ILD, cardiac involvement, PAH, dysphagia, cancer, ANA, anti-Jo-1 and anti-SSA positive at any time; muscle disease activity and global disease activity at disease onset; laboratory test results at diagnosis including low total protein (TP), low album (ALB), evaluated IgG, IgA, IgM, Creatine kinase (CK) at initial visit. There were no missing values among the prognostic factors in the data set.

### Disease course assessment

Disease course was defined as monocyclic, polycyclic, or chronic progress if at least 2 years of follow-up after diagnosis was available according to the criteria applied in previous studies [19,20]. Disease course was categorized as not assessed (NA) if there was less than 2 years of follow-up after diagnosis.

### Statistical analysis

Student's t tests or Mann-Whitney U tests were used for continuous variables and the χ^2 ^test was performed for categorical variables. Kaplan-Meier curves were used to illustrate the proportions of survival and the differences between groups were tested by using log-rank tests. The hazard ratios (HR) and 95% confidence intervals (CI) for death were calculated with Cox proportional-hazards models. In order to demonstrate possible significant explanatory risk factors for death, we first analyzed prognostic factors with the univariate Cox regression model. Variables with P ≦ 0.15 were considered possible confounders and retained in subsequent multivariate Cox proportional hazard analysis. All reported P values were two-sided. P values less than 0.05 were considered statistically significant. The statistical package used was SPSS software version 16.0 (SPSS Inc. Chicago, IL, USA).

## Results

### Clinical characteristics of IIM

188 patients were enrolled in the study. Among of them, 158 patients were available for muscle biopsies, while other 30 patients with dermatomyositis did not did not undergo muscel biopsies because of classical rash and muscle presentation. All cases biopsy were confirmed both by an experienced myopathologist and an experienced rheumatologist with particular interest in the disease. Clinical characteristics of 188 patients are shown in Table 1. The 188 patients were classified into 5 groups: DM (53.2%), PM (21.8%), OM (14.4%), ADM (6.9%), and JM (3.7%). Mean age at onset (± standard deviation) was 43.8 ± 15.8 years and male to female ratio was 1:2.1 in all patients. We observed females were five times more predominant than males in the OM subgroup, however, males were more predominant than females in the JM subtype. Among patients with OM, 15 (55.6%) had Sjögren's syndrome, 11(40.7%) systemic sclerosis, and 9(33.3%) rheumatoid arthritis, while only 2(7.4%) had primary biliary cirrhosis.

**Table 1:** Classification, demographics, clinical characteristics and treatment of patients with idiopathic inflammatory myopathies

	All	PM	DM	ADM	OM	JM	P value
**No. of patients no(%)**	188	41(21.8)	100(53.2)	13(6.9)	27(14.4)	7(3.7)	
Died	32	5	20	4	2	1	NA
Mortality (%)	17.0	11.9	20	30.8	7.69	14.3	NA
Male: female ratio	1:2.1	1:2.4	1:1.8	1:1.6	1:8	1:0.4	NA
Mean age ± S.D at onset (y)	43.8(15.8)	44.9(15.0)	44.9(14.9)	50 (13.8)	42(16.8)	15.7(2)	< 0.001†
Complicated with ILD, n, %	92(48.9)	17(40.5)	49(49.0)	13(100.0)	11(42.3)	2(28.6)	0.003
Complicated with PAH, n, %	20(10.6)	7(16.7)	7(7.0)	0(0.0)	6(23.1)	0(0.0)	> 0.05
Complicated with malignancy, n, %	11(5.9)	1(2.4)	10(10.0)	0(0.0)	0(0.0)	0(0.0)	> 0.05
Complicated with oropharyngeal dysphagia, n, %	75(39.9)	14(33.3)	36(36.0)	5(38.5)	15(57.7)	5(71.4)	> 0.05
Cardiac involvement, n, %	64(34.0)	14(33.3)	37(37.0)	4(30.8)	4(15.4)	5(71.4)	> 0.05
Mechanic hand at anytime, n, %	28(14.9)	1(2.4)	21(21.0)	3(23.1)	3(11.5)	0(0.0)	0.038
Raynaud phenomenon at anytime, n, %	29(15.4)	5(11.9)	10(10.0)	3(23.1)	11(42.3)	0(0.0)	0.001
Creatine kinase at initial visit in our center, mean (IQR)	1440.9 (83, 1561.8)	2106.8 (594.5, 2577.5)	1117.4 (68.3, 971.3)	67.9 (35, 93.5)	1867.9 (236, 2452)	3065.4 (114, 7908)	0.013††

† indicates that the comparison between PM, DM, ADM, OM, vs JM (p < 0.001), but the comparison among PM, DM, ADM and OM were no significant.

††indicates that the comparison between PM vs DM (p < 0.05), PM vs ADM (p < 0.01), DM vs JM (p < 0.05), ADM vs OM (p < 0.05), ADM vs JM (p < 0.01).

PM: polymyositis; DM: dermatomyositis; ADM: amyopathic dermatomyositis; OM: overlap myositis; JM: juvenile polymyositis or dermatomyositis; ILD: interstitial lung disease; PAH: pulmonary arterial hypertension; IQR: interquartile range; NA: not assessment.

The lung was the most common extramuscular target in our cohort patients (Table 1). The overall frequency of ILD was 48.9%. 16 patients concurred and 76 patients developed ILD within an average 2.1 years (range: 0.07 to 18 year) after disease onset. All ADM patients in our study presented with ILD, while JM patients presented with less ILD (2 out of 11 patients). 20 patients (10.6%) developed PAH after disease onset. PAH was distributed in OM (n = 7, 35%), DM (n = 7, 35%), PM (n = 6, 30%) patients, but not found in ADM and JM patients. 64(34%) patients exhibited cardiac involvement only, with subclinical electrocardiogram (ECG) changes mainly including sinus tachycardia, ST-T changes, or arrhythmia.

It was noted that 11 patients (5.9%) had malignancies including three lung cancers, two breast cancers, one ovarian cancer, one hepatic cancer, one leukemia, one esophageal carcinoma, one malignant thymoma and one nasopharyngeal carcinoma. Most of the malignancies (90.9%) were associated with DM; only one cancer patient was suffering with PM. The three lung cancers patients were treated by surgery or chemotherapy with Carboplatin and VP-16 which were not beneficial for myositis, patients with breast cancer, esophageal carcinoma, malignant thymoma, hepatic cancer and nasopharyngeal carcinoma were only done surgery, patients with leukemia were used chemotherapy with Hydroxycarbamide which was not beneficial for myositis.

### Clinical differences between surviving and non-surviving patients

Table 2 demonstrates the difference in demographic characteristics of the surviving and non-surviving groups during follow-up. There were statistically significant differences with regard to mean age at disease onset, presence of malignancy, cardiac involvement, low TP level and ALB levels at diagnosis, anti-Jo-1 and anti-SSA positive (p = 0.000, 0.000, 0.023, 0.044, 0.038, 0.016, 0.048, respectively).

**Table 2:** Demographic characteristics of survival and non-survival groups during follow-up

Variables	Non-survival group	Survival group	P values
**No. of patients (%)**	32(17.0)	156(83.0)	
**Sex, no. (%)**			
Male: female ratio	01:01.9	01:02.1	0.837
Classification of diagnosis			
PM	5(15.6)	37(23.7)	0.363
DM	20(62.5)	80(51.3)	0.331
ADM	4(12.5)	9(5.8)	0.241
OM	2(6.25)	24(15.4)	0.261
JM	1(3.13)	6(3.9)	1
Mean age ± S.D at onset (year)	52.9 ± 15.8	41.9 ± 15.2	**0**
Disease duration from onset to diagnosis, median (IQR) year	3.8(2, 12.8)	6.5(3, 12)	0.215
Skin ulcers, (%)	6(18.8)	15(9.6)	0.212
Complicated with ILD at disease course, (%)	17(53.1)	75(48.1)	0.558
Complicated with PAH at disease course, (%)	3(9.4)	17(10.9)	1
Complicated with malignancy, (%)e	7(21.9)	4(2.6)	**0**
Complicated with dysphagia at disease course, (%)	8(25.0)	67(42.9)	0.074
Cardiac involvement at disease course, (%)	17(53.1)	47(30.1)	**0.023**
Raynaud phenomenon at disease course, (%)	4(12.5)	25(16.0)	0.79
Low TP level† at diagnosis, (%)	10(31.3)	24(2.6)	**0.044**
Low ALB levels† at diagnosis, (%)	16(50.0)	46(29.5)	**0.038**
Elevated IgG†† at diagnosis, (%)	8(25)	47(30.1)	0.672
Elevated IgA†† at diagnosis, (%)	9(28.1)	25(15.4)	0.122
Elevated IgM†† at diagnosis, (%)	5(15.6)	20(12.8)	0.775
Creatine kinase at initial visit in our center, median (IQR)	260.5 (71.8, 1173.8)	491.5 (83.3, 1613.5)	0.489
Autoantibodies positive at disease course, (%)			
ANA positive	16(50.0)	85(54.5)	0.699
Anti-RNP positive	4(12.5)	13(8.3)	0.497
Anti-Jo-1 positive	0(0.0)	24(15.4)	**0.016**
Anti-SSA positive	2(6.3)	33(21.2)	**0.048**
MYOACT global disease activity, 10-cm VAS score at disease onset	6.21 ± 2.61	6.64 ± 2.60	0.408
MYOACT muscle disease activity, 10-cm VAS score at disease onset	7.09 ± 1.34	6.91 ± 1.3	0.47
MYOACT, subscale VAS score > 0 at disease onset			
Constitutional	25(78.1)	129(82.7)	0.614
Cutaneous	25(78.1)	111(71.2)	0.518
Skeletal	10(31.3)	42(26.9)	0.666
Gastrointestinal	9(28.1)	57(36.5)	0.421
Pulmonary	4(12.5)	12(7.7)	0.483
Cardiac	9(28.1)	35(22.4)	0.497

†refers to values that are below the lower limit of normal laboratory assay standards.

††refers to values that are higher than the upper limit of normal laboratory assay.

ANA, antinuclear antibody; RNP, ribonucleoprotein; SSA: Sjögren's syndrome antigen.

### Treatment and Clinical course

The therapies received by patients in our patients were varied according to disease severity, experience of treating physician and changing treatment strategies. All patients received corticosteroid at a dose between 0.5 mg/kg and 1 mg/kg as part of their initial therapy after suspecting a diagnosis of IIM. Meanwhile, because of the concerns about steroid side-effects, the well improved response to immunosuppressants with earlier administration and personalized treatment, over three-quarters of our patients (145/188, 77.1%) also received at least one or more of immunosuppressants including methotrexate (83/145, 57.2%), cyclophosphamide (87/145, 60%), azathioprine (62/145, 42.8%), intravenous immunoglobulin (IVIG) (31/145, 21.4%), hydroxychloroquine (17/145, 11.7%) or mycophenolate mofetil (18/145, 12.4%).

In 166 patients with a follow-up time of at least 2 years, the disease course was monocyclic in 25 patients (15.1%), polycyclic in 132 patients (79.5%) and chronic progressive in 9 patients (5.4%). We further compared the difference in disease course in surviving and non-surviving patients. The results showed the polycyclic course predominated in surviving patients (p = 0.000), while the chronic progressive course mainly existed in non-surviving patients (p = 0.017).

### Mortality and prognostic factors for death

**Figure 1:**
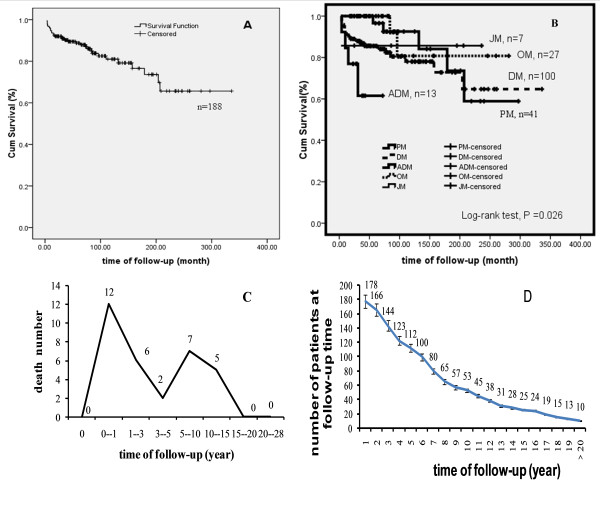
**Kaplan-Meier estimate of survival of 188 Chinese Han population patients with IIM, and stratified according to the type of myopathy**. **A**. Survival curves of patients with IIM 1 year survival rate is 93.6%, 5 year survival rate is 88.7%, 10 year survival rate is 81.0%, 15 year survival rate is 73.6%, 20 year survival rate is 65.6%; **B**. Survival curves stratified according to the subtypes of IIM. **C**. Death number within entire follow-up period. The mortality of IIM patients in China was characterized with double hump-like shape. D. shows a plot of the total number of patients at follow-up time. > 20 indicate the time of follow-up is over 20 years.

The mean follow-up time was 7.5 years. Disease-related deaths occurred in 17% of all patients. The cumulative survival estimate was 93.6%, 88.7%, 81%, 73.6% and 65.6% for 1, 5, 10, 15, and 20 years, respectively (Figure 1A). Survival analysis for further clinicopathologic subgroups revealed that patients with various forms of IIMs have different survival rates (Figure 1B).

Although death events have been observed at 1-15 years after disease diagnosis, a bimodal overall mortality distribution of IIM was established; with an initial peak at around the first 3 years (eighteen of the 32 deaths) and a second smaller peak occurring in 5-15 years (twelve of the 32 deaths) after disease diagnosis (Figure 1C). Figure 1D shows a plot of the total number of patients according to the follow-up time.

To maximize and identify the independent predictors for mortality, we first analyzed prognostic factors with the univariate Cox regression model in which variables with P ≦ 0.15 were considered possible confounders and retained in subsequent multivariate Cox proportional hazard analysis. The univariate analysis revealed that age at disease onset (p < 0.001), skin ulcers (p = 0.01), cardiac involvement (p = 0.11), presence of cancer (p < 0.001) or ILD (p = 0.12), low TP level (p = 0.15), low ALB level (p = 0.02), and elevated IgA at diagnosis (p = 0.08) were likely to be the risk factors for mortality. Meanwhile, dysphagia (p = 0.11), myalgia (p = 0.01) and anti-SSA positive at any time (p = 0.11) were shown to be possible protective factors. Because there could be interactions among these factors, we further confirm these predicators with multivariate analysis. The independent predictors for mortality were age at disease onset [hazard ratio (HR):1.05, 95% CI 1.02-1.08], the presence of cancer (HR:3.68, 95%CI 1.39 - 9.74), and elevated IgA level at diagnosis (HR:2.80, 95% CI 1.16, 6.74).

### Long-term outcomes in surviving patients

156 surviving patients were personally reexamined after a mean follow-up of 8.1 years (range 1.42-28 years). 115 (73.7%) patients had a MDI total score of > 0. Damage occurred most frequently in the pulmonary system, followed by the skeletal, endocrine, muscular and cutaneous systems (Table 3).

**Table 3:** Frequency of damage in the IIM patients at the end of the follow-up using the Myositis Damage Index (MDI)

Damage item	Survival IIM patients‡, n = 156, (%)
Muscle severity, VAS	31(20.0)
Muscle atrophy	7(4.7)
Weakness	53(34.0)
Muscle dysfunction	10(6.7)
Skeletal severity, VAS, no of test(149)^#^	44(29.4)
Joint contracture	1(0.7)
Osteopososis with fracture	4(2.8)
Avascular necrosis	5(3.5)
Arthropathy	9(6.3)
Cutaneous severity, VAS	31(20.0)
Calcinosis	7(4.7)
Alopecia	2(1.3)
Cutaneous scarring	15(9.3)
Poikiloderma	20(12.7)
Lipodystrophy	4(2.6)
Gastrointestinal severity, VAS	8(5.3)
Dysphagia	8(5.3)
Gastrointestinal dysmotility	1(0.7)
Gastrointestinal infarction	1(0.7)
Pulmonary severity, VAS	77(49.3)
Dysphonia	11(7.1)
Impaired lung function	75(48.1)
Pulmonary fibrosis	75(48.1)
Pulmonary hypertension	20(12.8)
Cardiovascular severity, VAS	27(17.3)
Hypertension	26(16.7)
Ventricular dysfunction	2(1.3)
Angina	3(1.9)
Myocardial infarction	3(1.9)
Peripheral vascular severity, VAS	2(1.3)
Tissue pulp loss	0(0.0)
Digit loss	2(1.3)
Thrombosis	0(0.0)
Claudication	0(0.0)
Endocrine severity, VAS	41(26.3)
Growth failure	0(0)
Delay in secondary sexual characteristics	0(0)
Hirsutism	0(0.0)
Irregular menses	2(1.3)
Amenorrhea	0(0.0)
Diabetes	41(26.3)
Infertility	NA
Sexual dysfunction	NA
Ocular severity, VAS	4(2.7)
Cataract	2(1.3)
Vision loss	1(0.6)
Infection severity, VAS	4(2.6)
Chronic infection	3(2.0)
Multiple infections	1(0.6)
Malignancy severity, VAS	4(2.6)
Malignancy	4(2.6)
Total damage	
Total MDI severity of damage > 0†	112(71.8)
Total MDI extent of damage > 0‡	115(73.7)

#indicates the number after excluded the patients complicated with rheumatoid arthritis (n = 7) NA: not applicable. VAS, visual analogue scale.

† Severity of damage was assessed in 9 organ systems and 2 categories of illness (infection and malignancy) and summed to provide a total MDI severity of damage score.

‡ Individual damage items were assessed in each organ system and category of illness and summed to provide a total MDI extent of damage score.

Table 4 shows the physical status, functional ability, disease activity and drug use status of survival patients at the end of the follow-up. 85.9% of the patients had no disease activity, as shown by global disease activity 10-cm VAS score = 0. Only 14.1% of the patients had a score > 0. Twelve (7.7%) patients exhibited muscle disease activity that defined with muscle disease activity 10-cm VAS score > 0.2. As measured by the HAQDI, 130 patients had no disability at all, and 26 patients had mild to moderate disability. Similar results were obtained by MRS with regard to disability.

**Table 4:** Disease status and drug use status of survival patients with IIM through using the MDAAT, HAQDI and MRS at the end of follow-up.

	All	PM	DM	ADM	OM	JM
**No. of patients (%)**	156	37	80	9	24	6
MYOACT global disease activity, 10-cm VAS score = 0, n. (%)	134(85.9)	31(83.8)	69(86.3)	7(77.8)	21(87.5)	6(100.0)
MYOACT global disease activity, 10-cm VAS score > 0, n. (%)	22(14.1)	6(16.2)	11(13.8)	2(22.2)	3(12.5)	0(0.0)
MYOACT muscle disease activity, 10-cm VAS score > 0.2, n. (%)	12(7.7)	3(8.1)	6(7.5)	2(22.2)	1(4.2)	0(0.0)
MYOACT, subscale VAS score > 0.2, no (%).						
Constitutional	13(8.3)	1(2.7)	9(11.3)	1(11.1)	2(8.3)	0(0.0)
Cutaneous	11(7.1)	1(2.7)	6(7.5)	2(22.2)	2(8.3)	0(0.0)
Skeletal	7(4.5)	4(10.8)	2(2.5)	0(0.0)	1(4.2)	0(0.0)
Gastrointestinal	0(0.0)	0(0.0)	0(0.0)	0(0.0)	0(0.0)	0(0.0)
Pulmonary	15(9.6)	6(16.2)	6(7.5)	2(22.2)	1(4.2)	0(0.0)
Cardiac	2(1.3)	2(5.4)	0(0.0)	0(0.0)	0(0.0)	0(0.0)
Patients with HAQDI score = 0, n. (%)	130(83.3)	33(89.2)	64(80.0)	8(88.9)	17(70.8)	6(100.0)
Patients with HAQDI score > 0, n. (%)	26(16.7)	4(10.8)	15(18.8)	1(11.1)	6(25.0)	0(0.0)
Patients with M RS = 0, n. (%)	131(84.0)	34(91.9)	66(82.5)	9(100)	16(66.7)	6(100.0)
Patients with MRS ≥1, n. (%)	25(16.0)	3(8.1)	14(17.5)	0(0.0)	8(33.3)	0(0.0)
Medication						
Off all drugs	29(18.6)	9(24.3)	16(20.0)	1(11.1)	3(12.5)	0(0.0)
Corticosteroid, 0-5 mg/d, n. (%)	73(46.8)	16(43.2)	37(46.3)	4(44.4)	12(50.0)	4(66.7)
Corticosteroid, 5-10 mg/d, n. (%)	34(21.8)	8(21.6)	17(21.3)	2(22.2)	5(20.8)	2(33.3)
Corticosteroid, 10-15 mg/d, n. (%)	9(5.8)	1(2.7)	6(7.5)	0(0.0)	2(8.3)	0(0.0)
Corticosteroid, > 15 mg/d, n. (%)	11(7.1)	3(8.1)	4(5.0)	2(22.2)	2(8.3)	0(0.0)
Second-line agents†, n. (%)	30(19.2)	10(27.0)	14(17.5)	1(11.1)	3(12.5)	2(33.3)

MYOACT, the Myositis Disease Activity Assessment Visual Analogue Scales; VAS, visual analogue scale; HAQDI, health assessment questionnaire disabled index; MRS, modified rankin scale.

†second-line agents include methotrexate, cyclophosphamide, azathioprine, intravenous immunoglobulin, hydroxychloroquine or mycophenolate mofetil.

29 patients (18.6%) were off all drugs after 4.5 years (range 0.7-14 years) of treatment, and they had been off drugs for an average of 4.1 years (range 0.5-15.2 years). 127 patients (81.4%) continued taking medications. 73 patients (46.8%) were taking corticosteroid with dosage of ≤5 mg/d, 34 patients (21.8%) were taking corticosteroid with dosage of 5 to 10 mg/d, 9 patients (5.8%) were taking corticosteroid with dosage of 10 to 15 mg/d, 11 patients (7.1%) were taking corticosteroid with dosage of > 15 mg/d, and 30(19.2%) patients were taking second-line agents.

## Discussion

This study was a mean 7.5 years follow-up of a large, single centre cohort of patients with IIM classified with PM, DM, ADM, OM and JM in China. To our knowledge, this is the first study to describe the long-term organ damage and physical health status in a large cohort of patients with IIM from China, utilizing standardized tools such as MDI, HAQDI and MRS. In this study, we have demonstrated that patients with IIM in China have good outcomes over long-term follow-up.

Few statistics on age at disease onset have been obtained directly in previous studies. Most studies reported age at diagnosis. In this retrospective cohort study, we described the relative "true" disease age through utilizing age at disease onset, although many of inflammatory myopathies have an insidious onset and early course which may lead difficult to establish a true onset.

Study on risk factors and survival of IIM patients with ILD has been reported from China. It was reported Chinese IIM patients tended to have high frequency of IIM-associated ILD [26]. The prevalence of ILD was higher in Chinese patients, Japanese patients and Arab Jordanian patient than Caucasian patients with IIM [27-30]. Indeed, about 50% of Chinese and Japanese patients with IIM develop ILD whereas only approximate 30% of Caucasian patients with IIM develop ILD [20,25-31]. High frequency of IIM-associated ILD may be a specific feature in eastern Asia.

An association between IIM and cancer has been widely reported in the literature. Several studies from Japan, European and American indicated that the overall prevalence of malignancy in IIM ranged from 9% to 40% [8-10,12,13,15,21,29,32-35]. However, our study showed very low prevalence of malignancy (5.9%), which is consistent with a study from Arab Jordan [27]. Indeed, our study was also similar to a recent finding from a nationwide cohort study of 1655 Chinese patients with PM/DM from Taiwan in which 128 patients (7.7%) had malignancies [36].

In various studies, mortality rates have varied from 10%-40% [1,8-11,13-15,17,18]. However, some of those previous investigations excluded overlapping, juvenile, ADM, or malignancy-associated cases, which are important subgroups of IIM. This retrospective study of 188 IIM patients is more representative of overall IIM patients. The overall mortality in our study was 17%, similar to other published series [11,18,31,37], but lower than that Troyanov Y reported [7]. Importantly, our results indicated that cumulative survival rate was better than other series reported over 15 years (Table 5) [7,9-12,15,29,32,33]. Our analyses show that high mortality occurred within 3 years, which was consistent with previous studies (Figure 1C) [8,9]. There was another death peak within 5 to 15 years in our IIM patients. In view of two significant peak of mortality within three years, and 5 to 15 years, we further studied the alteration after excluding patients with a follow-up of less 3 years and 10 years. Results showed that when patients with a follow-up of less than 3 years were excluded, the survival rate was 99.3%, 97.7%, 89.2%, 81.1% and 72.3% in 3, 5, 10, 15, and 20 years. Moreover, the 10-year, 15-year, and 20-year cumulative survival estimate of patients with a follow-up of over 10 years (n = 53) was 97.8%, 90.9%, 81%, and 73.6%, respectively. These data suggested that to improve the long-term outcome, continue three years of intensive outcome monitoring is recommended for patients with IIM.

**Table 5:** Survival of patients with IIM at previous reports and this study

First author, published year (Ref)	location	Follow-up	Mean follow-up	No. of patients	Clinical-pathologic subgroup	Survival rates (%)		
						1-year	5-year	> 5-year
Medsger 1971 [10]	USA	1947-1968	7 years	124	PM, DM, JDM, OM, CAM	72	65	53(7 yr)
Benbassat1985 [8]	Israel	1956-1976	20 years	92	PM, DM, JDM, OM, CAM	72	52	--
Hochberg1986 [11]	USA	1970-1981	8 years	76	PM, DM, JDM, OM	94.5	80.4	72.8 (8 yr)
Maugars 1996 [9]	France	1973-1998	--	69	PM, DM, JDM, OM, CAM	82.6	66.7	55.4 (9 yr)
Uthman 1996[31]	France	1980-1992	5.2 years	30	PM, DM, CAM, OM	--	89	85(10 yr)
Marie 2001 [12]	France	1983-1999	4 years	77	PM, DM, CAM	83	77	61 (15 y)
Sultan 2002 [1]	England	1978-1999	20 years	46	PM, DM, JDM, OM		95	83.8 (10 yr)
Dankó2004 [15]	Hungary	1976-2002	8.46 years	162	PM, DM, JDM, OM, CAM	95	92	89 (10 yr)
Troyanov2005[7]	France	1967-2001	8.7 years	100	PM. DM, CAM, OM	--	--	73(10 yr)
Airio. 2006 [32]	Finland	1965-1995	11 years	248 (176PM/72DM)	PM, DM	--	75(PM) 63(DM)	55(PM 53(DM)
Torres, et al 2006 [33]	Spain	1976-2005	9 years	107	PM, DM, JM, OM, OM, CAM	92	80	71(10 years)
Yamasaki Y, et al 2011[29]	Japan	1984-2010	---	197	PM, DM, ADM, CADM, OM	85	75	67(10 years)
Ours 2011	China	1986-2010	7.5 years	188	PM, DM, ADM, JM, OM, CAM	93.6%	88.7	81(10 years), 76.6(15 years) 65.6 (20 years)

Ref, reference; PM, polymyositis; DM, dermatomyositis; CAM, cancer associated myositis; OM, overlap myositis; JM, Juvenile PM/DM.

The predictors of mortality varied in different studies. Our study found presence of cancer and age at disease onset were associated with higher mortality, in agreement with published work [8-15,20]. Moreover, for the first time, elevated serum IgA at diagnosis was identified as an unexpectedly strong prognostic factor associated with mortality. An explanation for this may be that it seems to be related indirectly to the severe extent of muscle damage and might reflect the overall disease severity of inflammatory process. Several studies indicated that serum IgA antibodies in immune complexes are very effective at initiating a wide range of inflammatory responses, including phagocytosis, antibody dependent cellular cytotoxicity, oxidative burst, and cytokine release [38,39]. Inflammatory responses triggered by IgA are often mediated by the IgA-specific receptor FcαRI. A number of diseases including autoimmune diseases such as Sjogren's syndrome exhibit elevated levels of serum IgA. In the autoimmune disease Sjögren's syndrome, characterized by lymphoid cell infiltration of lacrimal and salivary glands, the N-glycans of monomeric IgA1 are oversialylated, which could presumably affect the FcαRI-IgA interaction and alter recycling of IgA [40]. The study suggested that elevated IgA in IIM may also trigger inflammatory responses in a manner similar to Sjögren's syndrome, which then resulted in muscle damage. However, this exact potential role of elevated IgA in the pathogenesis and outcome of inflammatory myopathies need other studies to elaborate in animal model and patient population.

Our study found that skin ulcer was a predictor for mortality in the univariate analysis (HR 3.17, 95% CI 1.27-7.93, p = 0.01), while it was not an independent predictor for mortality in the multivariate analysis. This is not consistent with a recent report from Japan which indicated that skin ulcer was an independent risk factor for prognosis [29]. There is still some debate on the relationship between skin ulcer and outcome. Several studies showed that skin ulcer was not associated with the risk of mortality in Chinese Han patients and Caucasian patients [8-10,13,15,17,20,33,32,37].

Previous studies from Japanese and Caucasian indicated that ILD was poor risk factor of IIM. Our study failed to identify several risk factors such as ILD, cardiac involvement, dysphagia that were often reported in previous studies. This may be explained as follows. First, there was no difference in the frequency of ILD and dysphagia found between non-survival and survival patients (Table 2). Second, we postulated another reason to be the early detection and aggressive therapy of these ILD patients. All of our patients with ILD received combinational treatment including corticosteroid and cyclophosphamide, azathioprine, IVIG, hydroxychloroquin or mycophenolate mofetil; and they responded to drugs well. In fact, there were two studies in Taiwan and France had found a similar result such that ILD and heart involvement did not influence the survival of DM/PM patients [25,28]. Third, dysphagia was improved in our study patients after diagnosis with treatment, which in turn helped to improve prognosis. Further, cardiac involvement in our study involved mainly subclinical features which may not influence the prognosis.

The disease duration from onset of signs to diagnosis was much longer than previous studies [9-12], however, the disease duration in our cohort study did not have effect on the prognosis, which may be explained in that some of our patients had already initiated corticosteroid therapy or immune-suppressed agents according to disease manifestation before the diagnosis of IIM. Our study also indicated that PM has better survival than DM (Figure 1B), which was very different from a number of other myositis mortality reports [9,15,29,33,32]. It is possible that the results are confounded by the inclusion of cancer-associated myositis within these clinical subgroups, rather than as a separate subgroup of patients.

The overall surviving patients in this study have favorable long-term outcomes assessed with MDI, MYOACT, HAQDI and MRS after a mean of 8.1 years of disease duration. Importantly, our findings suggested that surviving patients had less frequency of organ damage than in adult patients described by Rider LG et al [19]. Furthermore, it is noteworthy that only a small sizable proportion of the patients had persistently active disease at the end of the follow-up, as shown by the 7.7% or 14.1% frequency of abnormal muscle disease activity or MYOACT global disease activity scale, respectively. Disease activity was seen much more frequently in the pulmonary system, followed by constitutional symptoms such as fatigue, and skin complaints. Although the sample size of JM (6 survival patients at the end of follow-up) in this study was small, it is still noteworthy that all the surviving JM patients presented no active disease. Ravelli A et al demonstrated that persistently active disease was recorded in 41.2-60.5% of patients suffering juvenile dermatomyostis [17]. A similarly high frequency of persistently active juvenile dermatomyositis at the time of follow-up was reported in a Canadian multicenter [41]. The physical disability in our study was less than that described in others [17,20,41]. Likewise, patients with JM in our study had no physical disability at all. Overall, these findings showed marked improvement in functional outcome, disease activity and organ damage of IIM when compared with earlier literature at follow-up.

To minimize the bias of data, each data was collected by one physician, and the collected data was sufficient for use as a measure of IIM mortality and risk factors. Our data show that IIM spectrum in this study with regard to disease onset, clinical symptoms, prevalence of ILD, malignancy, partial prognostic factors is in general consistent with previous reports in Japan and European and American [8-15,20,26-29,35]. But the long-term outcome was better than previous studies over 15 years (table 5). These results both supported our data is validity. We speculated that favorable long-term outcomes of patients in this study could be attributed to the following: (i) positive screening of ILD, cardiac involvement, PAH, and serologic tests at any time during the clinical course. (ii) the prevalence of cancer was lower than others (5.9%). (iii) our patients received earlier administration, personalized and combined therapy. (iv) improvement in patients' recognition of the disease. (v) most patients (79.5%) were polycyclic course. (vi) differences in genetic and environmental backgrounds from other populations.

However, our study still does have some limitations. First, this series had a particularly low prevalence of JM (only seven cases in the whole study), which may have led to a tendency to overestimate the outcome of JM patients. Second, we did not obtain details of levels of muscle enzymes at disease diagnosis from all patients, which resulted in not analyzing the effects of skeletal muscle enzymes level on survival, because some patients were referred to our hospital. However, we still analyzed whether the serum CK level at initial visit at our center was associated with prognosis, results found there was no relation between CK level and prognosis. There is still some debate on the relationship between CK level and outcome. It was concluded that the level of CK did not reach any predictive value for mortality [28,32]. Third, the follow-up time was still relative short. A continued follow-up of our patients is under way. Another, organ damage was partly assessed by the MDI using telephone interview which may have resulted in an underestimation of the extent of damage or an inaccurate reflection of the severity of damage. Further prospective analyses need to be carried out with face to face interviews to examine the organ damage. Finally, we did not analyze the predicator factors associated with relapse and relapse frequency which may be beneficial for long-term outcome. So far, there is no any study about adult IIM patient reports the predicator factors associated with relapse and relapse frequency. However, one study has claimed that the persistence of Gottron's papules and nailfold abnormalities early in the disease course was associated with a longer time to remission in juvenile dermatomyositis [42]. Future works need to be done to claim the issues in adult patients with IIM.

## Conclusions

In conclusion, despite these limitations, the results of this study provide strong evidence that patients with IIM in Chinese Han populations had favorable functional outcomes and less organ damage at long-term follow-up. Those patients most likely to survive had a high chance of reaching stable disease status, and obtained long-term or possibly permanent remission to a large extent.

## Competing interests

The authors declare that they have no competing interests.

## Authors' contributions

XMS, GCW contributed to study design, data collection, statistical analysis, interpretation of data and drafting the manuscript; XL acquired the clinical data; YX acquired the serological data. All authors were involved in drafting the article or revising it critically for important intellectual content, and all authors read and approved the final manuscript.

## Pre-publication history

The pre-publication history for this paper can be accessed here:

http://www.biomedcentral.com/1471-2377/11/143/prepub
